# Perceived vibrato and the singing power ratio explain overall evaluations in opera singing

**DOI:** 10.3389/fpsyg.2025.1568982

**Published:** 2025-08-08

**Authors:** Haruka Kondo, Sotaro Kondoh, Shinya Fujii

**Affiliations:** ^1^Graduate School of Media and Governance, Keio University, Kanagawa, Japan; ^2^Faculty of Environment and Information Studies, Keio University, Kanagawa, Japan; ^3^Japan Society for the Promotion of Science, Tokyo, Japan

**Keywords:** voice, opera singing, overall score, vibrato, singing power ratio

## Abstract

In opera singing competitions, judges use an overall score to evaluate the singers' voices and determine their rankings. This score not only guides the singers' technique and expressiveness but also serves as a crucial indicator that can significantly influence their careers. However, the specific elements captured by this overall score remain unclear. To address this gap, the present study analyzed opera singing recordings to identify the factors that explain the overall score. Ten trained female Japanese singers performed “*Caro mio ben*” under standardized recording conditions. Four experts evaluated the recorded performances by assigning an overall score of 100 points and rating six vocal attributes: vibrato, resonance, timbre, diction, intonation, and expressiveness. The recordings were then analyzed to calculate specific acoustic and audio features, including the singing power ratio (SPR), harmonic-to-noise ratio (HNR), and loudness units full scale (LUFS). We developed two linear mixed models: the first regressed the overall score on the subjective vocal attributes, whereas the second predicted the overall score from the acoustic features. Evaluator identity was included as a random effect in both models. The results showed that vibrato was a significant predictor of the overall score in the first model. In the second model, only SPR emerged as a significant predictor. These findings suggest that vibrato, which reflects emotional expressiveness and vocal control, and SPR, which indicates the relative power in the high-frequency band (2–4 kHz) and assists a voice clearer than the accompaniment, are key factors in explaining the overall score in opera singing.

## 1 Introduction

In opera singing competitions, judges evaluate the performances and determine rankings. Although specific judgment criteria vary among competitions, many use scoring systems to assess the overall performance. For instance, in the vocal division of The Music Competition of Japan, each judge assigns a score out of 25 to the singer's overall performance, and the final ranking is determined by summing these scores after excluding the highest and lowest (The Music Competition of Japan Secretariat, 2024). In the International Vocal Competition Tokyo, judges allocate separate scores out of 50 for overall technique and expressiveness, and the combined total of 100 determines the ranking (International Vocal Competition TOKYO Guideline, 2024). A similar approach is used in the International Chopin Piano Competition, where participants receive an overall score (18th Chopin Competition Warsaw Rules, 2021). In all of these competitions, the overall score plays a pivotal role in determining rankings, which can have a substantial impact on musicians' careers. In vocal competitions, judges are typically selected based on their expertise in the field. Most have extensive professional experience, often spanning 10–40 years, including careers as vocal instructors, international performers, and faculty members at music conservatories or universities. Hence, their evaluations are considered to carry substantial weight in determining the final rankings. However, detailed judgment criteria are rarely disclosed, and evaluations inevitably reflect the judges' individual preferences and experiences. Consequently, it remains unclear which specific elements of a singer's voice contribute to the overall score.

Previous research has investigated the subjective attributes that contribute to superior singing evaluation. These attributes include the singing technique (Subotnik, 2003, 2004), perceived potential or talent based on voice (Davidson and Da Costa Coimbra, 2001; Hollien, 1993; Watts et al., 2003), and vocal quality (Geringer and Madsen, 1998). A survey of 1,000 vocal instructors identified vocal quality, intonation, and musicality as the most important factors (Watts et al., 2003). Notably, the study by Wapnick and Ekholm (1997) provides valuable insights into which subjective evaluation scale items might explain the overall score. In their research, experts repeatedly evaluated recorded singing performance, and the consistency of evaluations within and between judges was assessed. Their findings revealed correlations between the overall score and attributes such as vibrato, resonance, timbre, and diction. However, this study did not employ statistical modeling to determine which specific attributes could predict the overall score, leaving the underlying determinants of the overall score unclear.

In addition, because judges assign overall scores based on the sound of the voice, objective acoustic features are considered important in evaluations, particularly when assessments are based on audio recordings. Quantitative studies of subjective singing evaluations began in the 1920s (Seashore and Metfessel, 1925). Since then, researchers have investigated acoustic features that characterize high-quality voices and their correlation with subjective evaluations (Ekholm et al., 1998; Robison et al., 1994; Wapnick and Ekholm, 1997). One particularly important aspect of opera singing is the ability of the voice to resonate throughout a large hall without amplification (Sataloff, 2017; Sundberg, 1990). One key acoustic feature that supports this ability is the “singer's formant” (Bartholomew, 1934; Sundberg, 1990), a cluster of harmonics centered around ~2.5 kHz for male singers and 3.16 kHz for female singers (Bloothooft and Plomp, 1986). The singer's formant can be quantified using the Singing Power Ratio (SPR), which measures the harmonic balance of a voice by comparing the strongest harmonic peak in the 2–4 kHz range with that in the 0–2 kHz range (Omori et al., 1996). Higher SPR values, indicating a smaller difference in power between the 2–4 and 0–2 kHz ranges, are associated with a bright, ringing tone (Omori et al., 1996). Research has shown that trained and untrained singers can be distinguished based on SPR-related values (Watts et al., 2003). These results suggest that SPR likely plays a critical role in determining overall scores in opera singing.

Other indicators that may influence the overall score include the harmonic-to-noise ratio (HNR) and integrated loudness units full scale (LUFS). HNR measures the amount of periodic (harmonic) energy in the voice and serves as an indicator of voice clarity (Murphy et al., 2008; Qi and Hillman, 1997). A higher HNR signifies a lower noise level, which listeners typically perceive as a clearer voice (Ferrand, 2002). In this context, “noise” refers not to external recording artifacts, but to intrinsic aperiodic and nonlinear components of the voice, such as irregular vocal fold vibrations, turbulent airflow, or features associated with vocal pathology. Trained opera singers typically produce very little noise in their vocal outputs (Ikuma et al., 2022). Integrated LUFS is a standardized metric used in audio for normalization purposes. This metric is designed to reflect the long-term perceived loudness of an entire recording, rather than short-term fluctuations, and is therefore more closely related to the overall perceptual impression of the piece. Therefore, in addition to SPR, both HNR and LUFS are likely to influence the overall opera singing scores. In this study, we selected objective acoustic and audio metrics based on their ability to be consistently extracted from the entire recording. All three indices met this criterion as they could be applied to full-length waveforms. However, it remains to be determined which of these features best accounts for the overall scores.

This study aimed to identify the factors that contribute to the overall scores in opera singing. To this end, we recorded opera performances, obtained evaluations from expert judges, and collected both overall scores and ratings for the six vocal characteristics identified in previous research (Wapnick and Ekholm, 1997). We also extracted acoustic features, including the SPR, HNR, and LUFS. Two linear mixed models were constructed: the first examined the relationship between overall scores and subjective vocal characteristics, whereas the second used acoustic and audio features, SPR, HNR, and LUFS, as predictors of overall scores. LUFS was used mainly because the acoustic metric of SPL was not available. By integrating the results of these models, we aimed to clarify the key determinants of opera singing scores from both the subjective and objective perspectives.

## 2 Materials and methods

### 2.1 Participants

Ten female Japanese singers specializing in classical vocal music (mean age ± SD = 25.10 ± 4.41) participated in this study. All participants were either currently enrolled at a music university, had graduated from a music university, or had received equivalent professional training. Table 1 provides detailed information on the participants' voice types and years of vocal experience.

**Table 1:** Singers' voice type, age, and vocal experience.

**Singer's ID**	**Voice type**	**Age (years)**	**Vocal experience (years)**
1	Soprano Leggero	30	13.0
2	Soprano Leggero	24	12.0
3	Soprano Lirico	25	10.0
4	Soprano Lirico	22	5.0
5	Soprano Lirico	22	0.5
6	Soprano Lirico	20	1.5
7	Mezzo-Soprano	26	11.0
8	Mezzo-Soprano	24	6.0
9	Soprano Lirico	23	8.0
10	Soprano Lirico	35	0.5

The recordings of the ten singers were evaluated by four vocal instructors, all professional singers (four females; mean age ± SD: 47.75 ± 12.26 years, range: 35–61 years). Their professional musical careers and vocal teaching experience are summarized in Table 2. Prior to the experiment, all vocal instructors confirmed that they had no history of hearing impairment, and none of them reported any hearing difficulties.

**Table 2:** Judges' age, professional musical career, and vocal teaching experience.

**Judge's ID**	**Age (years)**	**Professional musical career (years)**	**Vocal teaching experience (years)**
1	40	20.0	17.0
2	35	15.0	10.0
3	61	37.0	28.0
4	55	33.0	25.0

Ethical approval for the study was obtained from the Research Ethics Committee of Keio University Shonan Fujisawa Campus (Approval Number: 441). All participants were thoroughly informed of the experimental procedures and written consent was obtained prior to the experiment.

### 2.2 Procedure and data acquisition

The participants completed vocal exercises in a soundproof room before singing the assigned musical piece. The recorded data were used for acoustic analysis, and a separate evaluation session was conducted in which judges assessed the performance based on predefined criteria.

#### 2.2.1 Procedure for singers

Recordings were conducted in a sound-isolated booth with interior dimensions of 1.60 × 1.60 × 2.12 m (W × D × H). The walls and ceiling were fitted with soundproof panels to minimize external noise, and the floor was covered with a tile carpet to reduce the impact noise and surface reflections. Although the room was not fully anechoic, these treatments created a controlled recording environment with low ambient noise and limited reverberations. This room was specifically designed with an elevated floor to prevent the transmission of footstep vibrations, and silencers were installed in the air conditioning and ventilation ducts to eliminate ambient noise.

Although opera singers are typically accustomed to performing in large, reverberant spaces, such as concert halls and auditoriums, the present recordings were conducted in a small, low-reverberation booth to minimize ambient noise and acoustic interference. While this setting did not replicate the acoustic conditions of typical performance venues, it was intentionally chosen to ensure precise and consistent measurement of vocal acoustic features under controlled conditions.

Before recording, the singers completed a questionnaire regarding their vocal experience. They were then given 10 min of vocal warm-up in the soundproof room to acclimatize to the recording environment. Following the warm-up, each singer performed the assigned piece, *Caro mio ben*, a cappella. Before singing, a starting pitch was provided using a digital piano. The singers used music sheets placed on a stand during their performance, rather than singing from memory. Each singer performed the piece only once. *Caro mio ben*, composed by Tommaso Giordani in 1859, was selected for its accessibility, manageable vocal range, and low technical difficulty, making it suitable for singers with varying levels of experience. In addition, this piece is commonly used by vocal students in Japan.

The recording setup included a computer (MacBook Retina 12-inch, 2017, macOS Monterey, Apple, Inc.) connected to an audio interface (M-TRACK 2X2M, M-AUDIO) and a microphone (AT2035, Audio-Technica). The microphone, which had a frequency response range of 20–20,000 Hz, was positioned 20 cm from the singer's mouth. The frequency response of the microphone is relatively flat between 200 Hz and 4 kHz, with a gradual roll-off below 200 Hz (Audio-Technica, 2008). Given that the lowest note sung in *Caro mio ben* was D4 (293 Hz), the influence of the frequency characteristics of the microphone on SPR measurements was considered minimal. Audio recordings were captured using the Audacity software (ver. 3.4.2), with a standardized sampling frequency of 192 kHz. The preamplifier level of the audio interface was fixed and held constant across all singers to ensure consistent input levels, enabling appropriate calculation of the LUFS. LUFS was used in this study because of the unavailability of SPL.

#### 2.2.2 Procedure for judges

The evaluation sessions were conducted in the same soundproof room used for recording. Audio recordings were played on a computer (MacBook Retina 12-inch, 2017, macOS Monterey, Apple) connected to headphones (HD280pro, SENNHEISER). Before the session, the judges adjusted the playback volume to ensure consistent listening conditions across all the recordings.

Before beginning the evaluations, the judges completed a questionnaire detailing their vocal experiences and professional careers. They then listened to the recordings of the 10 singers, presented in a randomized order, and evaluated the performances based on two criteria: (1) an overall score on a 100-point scale and (2) six vocal attributes—vibrato, resonance, timbre, diction, intonation, and expressiveness—rated on a 7-point Likert scale (1 = very low, 7 = very high). The playback volume was standardized across all judges, and the judges did not alter the volume after this initial setup to ensure consistency throughout the evaluation process. These attributes were selected based on the previous research by Wapnick and Ekholm (1997). The judges were all professional singers; therefore, we did not provide formal definitions of the vocal attributes. However, for clarity, the six vocal attributes are described as follows: resonance refers to vocal depth and richness, whereas timbre represents tonal qualities such as brightness and warmth. Vibrato is characterized by its regularity, rate, and extent. Diction reflects pronunciation clarity and intelligibility, whereas intonation reflects pitch accuracy and stability. Expressiveness captures the singer's ability to convey emotions, use dynamics, and shape phrases effectively.

### 2.3 Analysis

#### 2.3.1 Acoustic analysis

We analyzed the acoustic features of the entire recording (including both vowels and consonants) using three parameters: SPR, HNR, and LUFS. Praat software (version 6.3.10) and MATLAB (R2024a, Audio Toolbox, MathWorks Inc.) were used for this analysis (Boersma and Weenink, 2024). To ensure the accuracy of the acoustic analysis, the recorded audio files were preprocessed to isolate the sung portions of the performances. Non-singing segments such as pauses and breaths were excluded.

The SPR was calculated from the power spectrum (expressed in decibels, dB) obtained by applying a Fast Fourier Transform (FFT) with a window size of 1,024 points and a bandwidth of 4,000 Hz. From the spectrum, we extracted the highest-amplitude harmonic peak within the low-frequency band (0–2 kHz), defined as *Power*_Low_, and the highest-amplitude harmonic peak within the high-frequency band (2–4 kHz), defined as *Power*_High_. The SPR was computed as the difference between these two peak amplitudes (Omori et al., 1996).


(1) $$\begin{array}{c} SPR\left(\text{dB}\right)={Power}_{\text{High}}-{\text{Power}}_{\text{Low}} \end{array}$$

Since both *Power*_High_ and *Power*_Low_ are already expressed in dB, the subtraction directly yields the SPR without further logarithmic conversion.

HNR was calculated using the autocorrelation method implemented by Praat. This parameter quantifies the ratio of the harmonic energy to the noise energy in the voice signal (Fernandes et al., 2023). Because both the harmonic and noise components are expressed in dB, the HNR formula reflects the difference between two logarithmic magnitudes:


(2) $$\begin{array}{c} HNR\left(\text{dB}\right)={Power}_{\text{Harmonics}}{-Power}_{\text{Noise}} \end{array}$$

where *Power*_Harmonics_ represents the power of the harmonic component, and *Power*_Noise_ represents the power of the noise component. The analysis was conducted using a frame-based window and the average HNR was calculated across the entire performance.

The integrated LUFS was calculated using the “integratedLoudness” function in MATLAB's Audio Toolbox based on the ITU-R BS.1770-4 standard. Rather than using LUFS values as absolute indicators of loudness, the model considered them as relative differences across singers. This is given that LUFS reflects the loudness of the audio signals, not of the singers *per se*.

#### 2.3.2 Statistics

Given the limited number of participants, a linear mixed-effects model was used to account for inter-rater variability and model the crossed data structure in which each singer was evaluated by multiple judges (10 singers × 4 judges = 40 observations). The model was estimated using restricted maximum likelihood (REML), with judge identity included as a random intercept. Linear mixed-effects models are well-suited for small-sample designs and have been shown to produce valid statistical inferences under such conditions (Schielzeth and Forstmeier, 2009).

To analyze the effects of subjective vocal attributes and acoustic features on the overall scores, two linear mixed-effects models were constructed. To assess the normality of the residuals, Shapiro–Wilk tests were conducted for all models. The analyses were conducted using R software (version 4.4.2) with the lmerTest and lme4 packages (Bates et al., 2015; Kuznetsova et al., 2017), which facilitated linear mixed-effects modeling with *p*-value estimation. The marginal and conditional R-squared values were calculated using the partR2 package (Stoffel et al., 2021).

The first model examined the impact of six vocal attributes–resonance, timbre, vibrato, diction, intonation, and expressiveness–on overall scores. In this model, six vocal attributes were treated as fixed effects, and judge variability was included as a random effect. The model formula is as follows:


(3) $$\begin{array}{l} Overall\;impression\;score\;\sim \\ Resonance+Timbre+Vibrato+Diction+\\ Intonation+Expressiveness+(1|JudgeID)\; \end{array}$$

The second model investigated the contribution of three acoustic features–SPR, HNR, and LUFS–to the overall scores. In this model, acoustic features were treated as fixed effects, whereas judge variability was treated as a random effect. The formula for this model is as follows:


(4) $$\begin{array}{c} Overallimpressionscore\;\sim SPR+HNR+LUFS+\left(1|JudgeID\right) \end{array}$$

For both models, the significance level α was set at 0.05. The marginal R-squared values ($R_{m}^{2}$) represent the explanatory power of fixed effects alone, whereas the conditional R-squared values ($R_{c}^{2}$) account for the explanatory power of both fixed and random effects. The confidence intervals (CI) for $R_{m}^{2}$ and $R_{c}^{2}$ were estimated using 100 bootstrap iterations. The variance inflation factor (VIF) was calculated using the car package (Fox and Weisberg, 2019) to assess multicollinearity among the predictor variables. The Shapiro–Wilk test was conducted on the residuals of both linear mixed-effects models to evaluate the normality assumption. The results indicated that the assumption was satisfied for both the vocal-attribute model (*W* = 1.00, *p* = 0.40) and the acoustic-feature model (*W* = 0.97, *p* = 0.35).

## 3 Results

**Figure 1:**
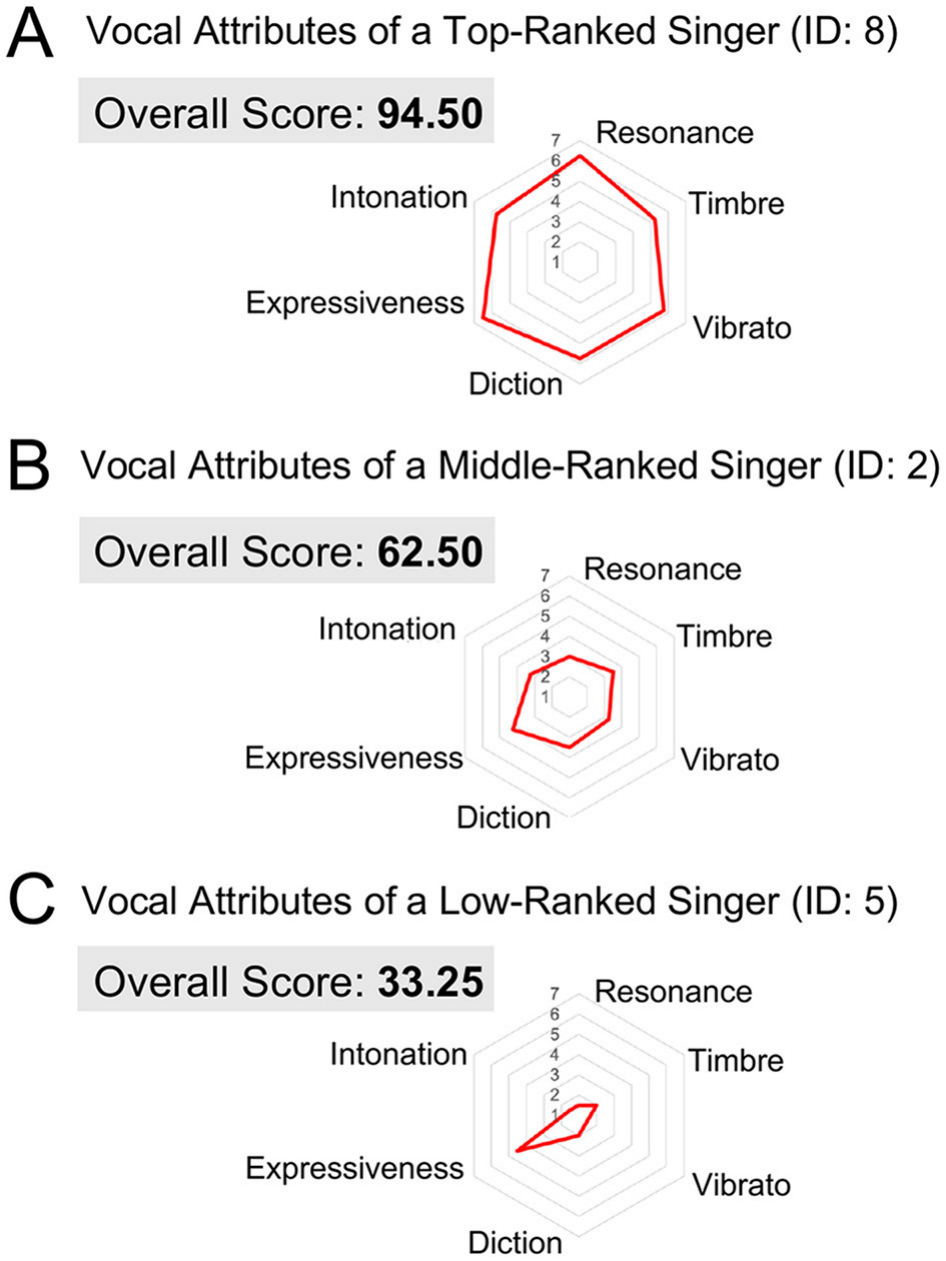
Vocal evaluation scores of three singers with different performance levels. **(A)** Vocal attributes of the top-ranked singer (ID: 8), who achieved the highest overall score. **(B)** Vocal attributes of the middle-ranked singer (ID: 2), who received a mid-range overall score. **(C)** Vocal attributes of the low-ranked singer (ID: 5), who obtained the lowest overall score.

**Figure 2:**
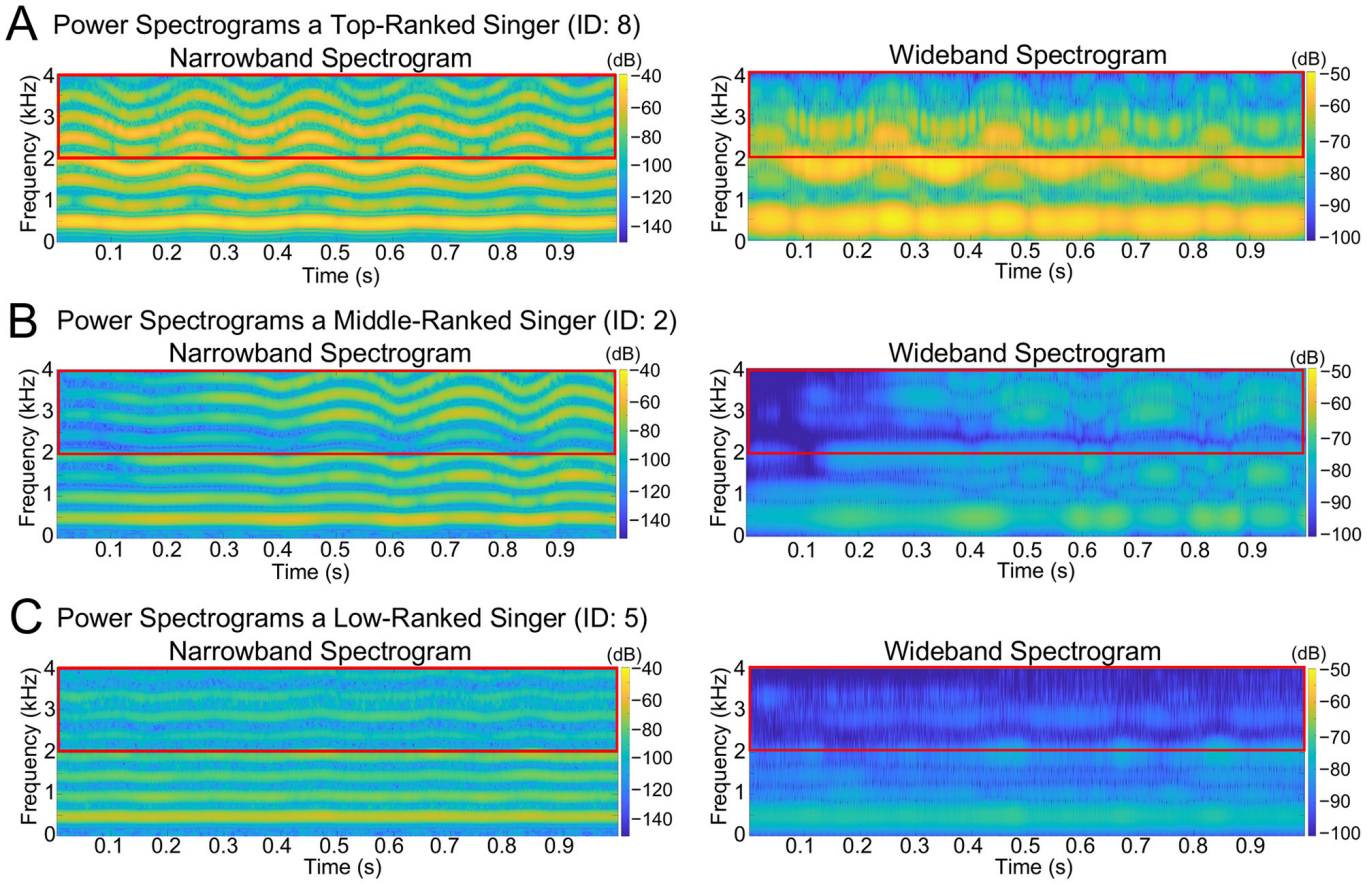
Spectrograms of three singers with different performance levels. **(A)** Power spectrograms of the singer with the highest overall score (ID: 8). **(B)** Power spectrograms of the singer with the mid-range overall score (ID: 2). **(C)** Power spectrograms of the singer with the lowest overall score (ID: 5). The left panels show narrowband spectrograms, and the right panels show wideband spectrograms, both derived from a B-flat note from the climactic phrase of *Caro mio ben*. The red horizontal box in each panel indicates the 2–4 kHz frequency range, which corresponds to the SPR band. For the narrowband spectrogram, we used a fixed window length of 2,048 samples, and for the wideband spectrogram, we used a short window length of 576 samples. Both analyses employed a Hamming window with 90% overlap and an FFT size of 4,096 points.

As representative examples, Figures 1 and 2 present the evaluation scores and spectrograms for the three singers who received different overall scores. The top-level singer (ID: 8) received the highest score, the middle-level singer (ID: 2) received a mid-range score, and the low-level singer (ID: 5) received the lowest score. Spectrograms were generated from each singer's highest-pitched note (B-flat) in *Caro mio ben*. In Figures 1A–C, the radar charts display the overall scores, along with the six vocal evaluation scores for each singer. Figures 2A–C show the corresponding narrowband (left) and wideband (right) power spectrograms. Narrowband spectrograms illustrate harmonic structures and vibrato modulations, while wideband spectrograms emphasize formant clusters and spectral energy distribution.

First, the top-ranked singer (ID: 8) achieved an overall score of 94.50 (Figure 1A) and displayed consistently high ratings across all the six vocal evaluation criteria. As shown in Figure 2A, the spectrogram featured prominent energy in the 2–4 kHz range associated with the singer's formant, and the B-flat note was performed with a regular vibrato. Second, the middle-ranked singer (ID: 2) received an overall score of 62.50 (Figure 1B), exhibiting moderate ratings across the six vocal attributes. The power spectrogram (Figure 2B) shows weaker energy in the 2–4 kHz band compared with the top singer. This singer applied vibrato to the B-flat note, but with wider pitch variation, fewer oscillations, and greater irregularity than those observed in the top-ranked singer (Figure 2A). Third, the low-ranked singer (ID: 5) obtained the lowest overall score of 33.25 (Figure 1C), reflecting low ratings across all six vocal attributes. The spectrogram (Figure 2C) indicates a very weak energy in the 2–4 kHz range and the absence of vibrato in the B-flat note. Individual ratings for the overall scores and six vocal attributes are provided in Table 3, and SPR, HNR, and LUFS are shown in Table 4.

**Table 3:** Individual ratings from four judges for each vocal attribute and singer.

**Judge's ID**	**Singer's ID**	**Overall score**	**Vibrato**	**Resonance**	**Timbre**	**Diction**	**Intonation**	**Expressiveness**
1	1	80	5	6	6	7	7	6
	2	65	4	3	3	3	4	2
	3	60	1	4	4	7	7	4
	4	70	5	2	2	3	7	3
	5	20	1	1	1	1	7	1
	6	60	4	3	3	3	6	5
	7	100	5	6	6	6	7	6
	8	100	6	6	6	7	7	6
	9	70	6	4	4	6	6	6
	10	50	2	3	4	2	4	5
2	1	80	6	6	6	7	7	2
	2	60	3	1	1	3	5	3
	3	40	3	3	2	3	6	2
	4	40	2	5	2	4	3	3
	5	20	1	1	2	3	6	1
	6	40	1	2	2	3	2	5
	7	100	3	6	7	7	7	6
	8	100	6	7	3	7	7	6
	9	50	4	2	2	5	6	6
	10	50	3	4	4	2	3	3
3	1	80	5	6	5	5	5	4
	2	75	4	5	5	3	4	4
	3	70	3	5	5	2	4	2
	4	60	3	2	1	1	4	3
	5	58	2	2	2	1	2	2
	6	68	3	3	4	2	3	3
	7	90	5	6	5	5	5	6
	8	78	4	5	5	2	5	4
	9	85	5	6	5	4	5	6
	10	50	3	4	3	1	4	3
4	1	85	5	6	6	6	5	7
	2	50	2	3	5	5	4	3
	3	50	3	4	5	5	3	4
	4	50	4	4	5	4	5	5
	5	35	2	3	3	3	3	2
	6	60	5	4	6	3	5	5
	7	65	4	5	6	6	4	6
	8	100	7	7	7	7	7	7
	9	75	5	5	5	7	6	5
	10	25	2	3	3	1	3	2

**Table 4:** Calculated acoustic features from each singer's recorded voice.

**ID**	**SPR (dB)**	**HNR (dB)**	**LUFS (dB)**
1	−10.76	47.30	−29.01
2	−24.65	53.11	−30.89
3	−14.10	48.38	−27.12
4	−19.81	44.61	−24.54
5	−29.75	53.26	−37.18
6	−22.30	50.81	−27.72
7	−22.08	48.39	−22.72
8	−15.64	44.59	−27.49
9	−17.71	46.01	−29.20
10	−17.99	43.79	−23.34

### 3.1 Effects of vocal attributes on overall scores

**Figure 3:**
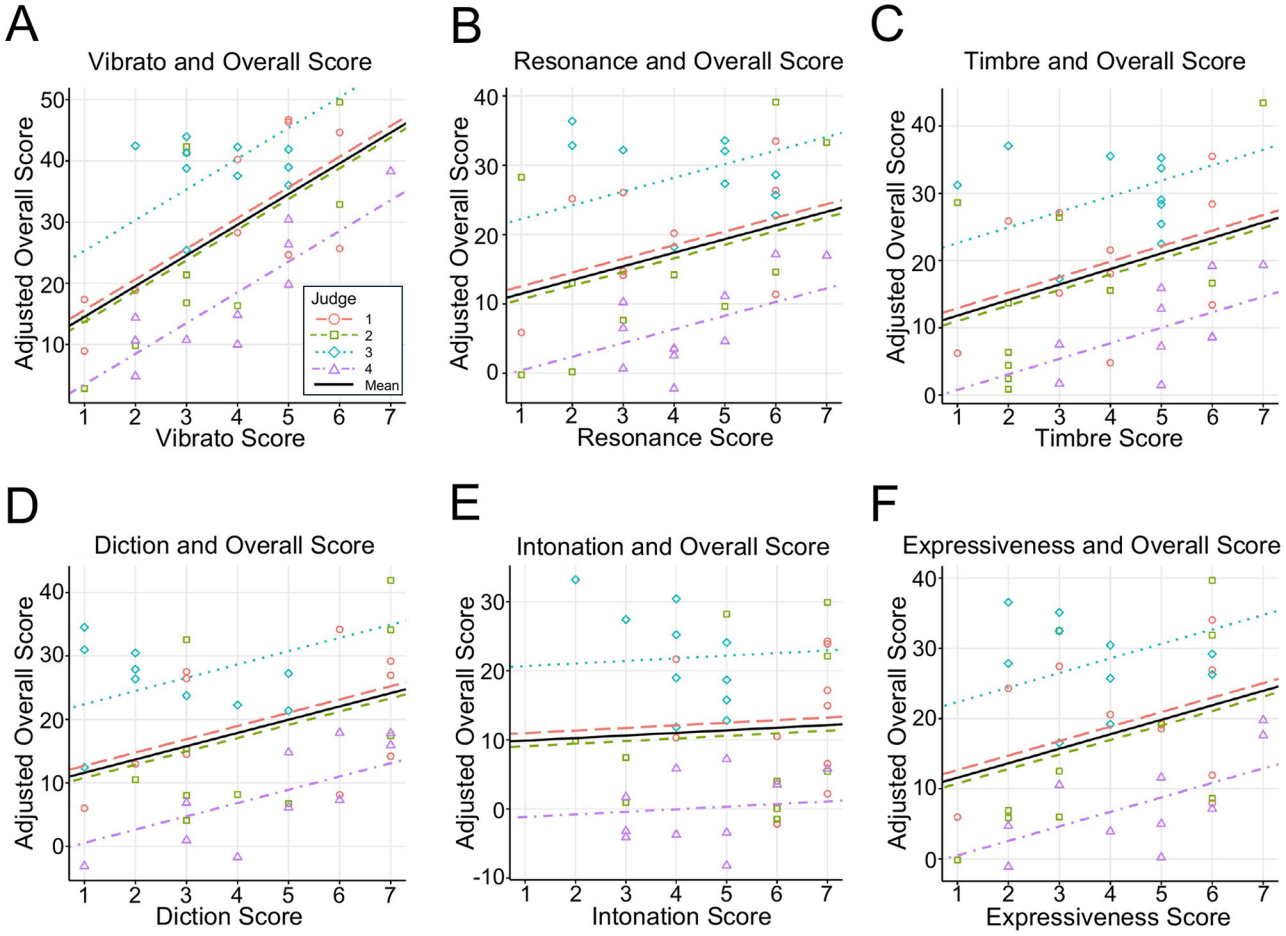
Scatter plots of vocal attributes vs. adjusted overall scores. Data were fitted using a linear mixed-effects model, where vocal attributes were treated as fixed effects and judge ID was included as a random effect. **(A)** Vibrato score plotted against the overall score. The adjusted overall score was calculated by subtracting β (resonance) × resonance score, β (timbre) × timbre score, β (diction) × diction score, β (intonation) × intonation score, and β (expressiveness) × expressiveness score from the original overall score. **(B–F)** Scatter plots of resonance, timbre, diction, intonation, and expressiveness scores were plotted against the adjusted overall score. In each case, the adjusted overall score was computed similarly by excluding the contribution of the other attributes from the original overall score.

The results from the linear mixed-effects model (Equation 3) assessing the influence of vocal attributes on the overall scores are summarized in Table 5. Among the six vocal attributes, vibrato had a significant positive effect on the overall scores (β = 5.02, *p* = 0.003; Figure 3A). By contrast, resonance (β = 1.97, *p* = 0.328; Figure 3B), timbre (β = 2.31, *p* = 0.192; Figure 3C), diction (β = 2.09, *p* = 0.176; Figure 3D), intonation (β = 0.38, *p* = 0.806; Figure 3E), and expressiveness (β = 2.06, *p* = 0.126; Figure 3F) were not statistically significant. All VIFs were below 5 (range = 1.84–4.85), indicating that multicollinearity was unlikely to severely bias parameter estimates. Although a VIF above 1 reflects some shared variance, values under 5 are generally considered acceptable in previous behavioral and acoustic research (Kutner et al., 2004; O'brien, 2007).

**Table 5:** Estimation of linear mixed-effects models fitted to overall score (fixed effects: vocal attributes).

**Variable**	**β**	***SE***	***df***	***t*-value**	***p*-value**	***VIF***
Vibrato	5.02	1.55	30.50	3.24	0.003^*^	2.55
Resonance	1.97	1.99	31.95	0.99	0.328	4.85
Timbre	2.31	1.73	32.10	1.33	0.192	3.30
Diction	2.09	1.51	32.40	1.38	0.176	3.62
Intonation	0.38	1.52	32.17	0.25	0.806	1.88
Expressiveness	2.06	1.31	30.20	1.57	0.126	2.14

Asterisk (^*^) shows statistical significance. Marginal *R*^2^ = 0.71; Conditional *R*^2^ = 0.84.

The marginal *R*^2^ ($R_{m}^{2}$) was 0.71 (95% CI: 0.55–0.86), and the conditional *R*^2^ ($R_{c}^{2}$) was 0.84 (95% CI: 0.75–0.91). These results indicate that fixed effects (subjective evaluation criteria) accounted for ~71% of the variance in the overall scores ($R_{m}^{2}$), and the full model, including both fixed effects and judge-level random intercepts, accounted for ~84% of the variance ($R_{c}^{2}$). The difference between $R_{m}^{2}$ and $R_{c}^{2}$ suggests that a random effect—specifically, variability among judges—contributes to the overall variance in scores.

### 3.2 Effects of acoustic features on overall scores

**Figure 4:**
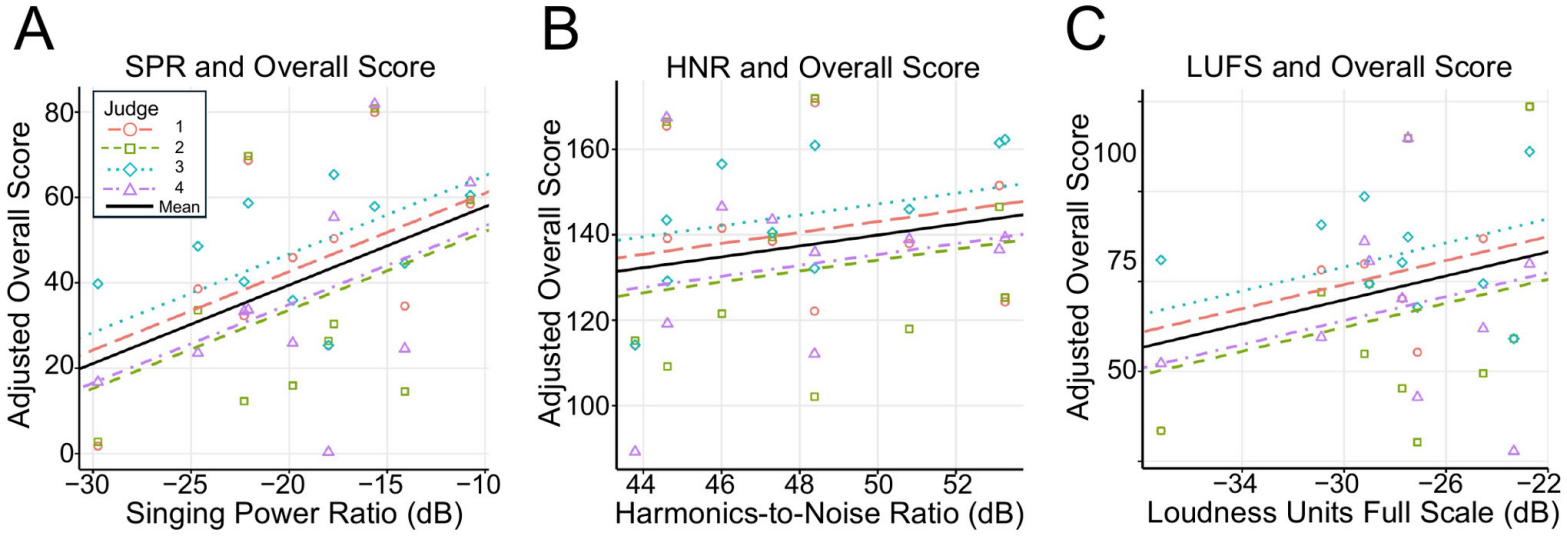
Scatter plots of acoustic features vs. adjusted overall scores. Data were fitted using a linear mixed-effects model, where acoustic features were treated as fixed effects and judge ID was included as a random effect. **(A)** Singing Power Ratio (dB) plotted against the adjusted overall score. **(B)** Harmonics to Noise Ratio (dB) plotted against the adjusted overall score. **(C)** Loudness Units Full Scale (dB) plotted against the adjusted overall score. In each case, the adjusted overall score was computed by excluding the contributions of the other acoustic features from the original overall score.

The results of the linear mixed-effects model (Equation 4) assessing the influence of acoustic features on overall scores are summarized in Table 6. Among the three acoustic features, SPR had a significant positive effect on the overall impression scores (β = 1.84, *p* = 0.034; Figure 4A). In contrast, HNR (β = 1.27, *p* = 0.44; Figure 4B) and LUFS (β = 1.34, *p* = 0.24; Figure 4C) did not exhibit statistically significant effects. All VIF values were below 5.

**Table 6:** Estimation of linear mixed-effects models fitted to overall score (fixed effects: acoustic features).

**Variable**	**β**	***SE***	***df***	***t*-value**	***p*-value**	***VIF***
SPR	1.84	0.83	36.00	2.21	0.034^*^	1.83
HNR	1.27	1.63	36.00	0.78	0.44	2.75
LUFS	1.34	1.12	36.00	1.20	0.24	1.92

Asterisk (^*^) shows statistical significance. Marginal *R*^2^ = 0.20; Conditional *R*^2^ = 0.20.

The $R_{m}^{2}$ was 0.20 (95% CI: 0.067–0.385), and the $R_{c}^{2}$ was 0.20 (95% CI: 0.078–0.398). There was little difference between $R_{m}^{2}$ and $R_{c}^{2}$ values.

## 4 Discussion

This study aimed to identify the key factors influencing the overall evaluation of opera singing. To achieve this, we recorded the performances of the classical Italian song *Caro mio ben* sung by trained vocalists, collected the overall scores and ratings for six vocal attributes, and analyzed the acoustic features of the recordings. Two linear mixed models were constructed: the first examined the relationship between overall scores and subjective vocal characteristics, while the second predicted overall scores based on acoustic features of SPR, HNR, and LUFS.

### 4.1 Effects of vocal attributes on overall scores

When we regressed the overall scores on subjective vocal characteristics, vibrato emerged as the only factor that showed a significant positive association with the overall scores (Figure 3; Table 5). This finding suggests that judges may place particular emphasis on vibrato when evaluating opera performance. By contrast, resonance, timbre, diction, intonation, and expressiveness did not show statistically significant effects.

#### 4.1.1 Vibrato

A previous study by Wapnick and Ekholm (1997) used Pearson's correlation coefficient to examine the relationship between overall scores and other vocal performance assessments. Their findings revealed a strong correlation between overall scores and vibrato ratings as well as consistency in judges' evaluations of vibrato. Similarly, our results indicate that vibrato ratings can predict overall scores.

Calculated vibrato is widely recognized as a key feature of opera singing, contributing to both vocal expressiveness and technical proficiency (Howes et al., 2004). A previous study comparing professional opera singers with students found that vibrato quality and control—rate and extent—were closely linked to singing proficiency (Amir et al., 2006). Since many judges and vocal instructors assess vibrato quality and control as indicators of advanced vocal techniques, vibrato is expected to play a crucial role in determining the overall opera performance scores.

As our main acoustic analysis focused on overall acoustic measures, such as SPR, HNR, and LUFS, we did not initially compute the vibrato-specific acoustic parameters. This was because our primary aim was to predict the judges' overall scores based on acoustic features calculated from the entire performance, whereas vibrato analysis typically requires localized examination of sustained pitch segments. However, given that vibrato emerged as the only significant predictor among the subjective rating items in our perceptual model, we conducted an exploratory analysis to examine whether this subjective vibrato score corresponded to objectively measurable features related to vibrato. Specifically, we examined whether perceived vibrato ratings could be predicted from two established acoustic parameters of vibrato: vibrato rate and vibrato extent (Sundberg, 1995) (see Supplementary methods). From each performance, a single sustained note was isolated, and both the vibrato rate and vibrato extent were calculated. The perceived vibrato rating was significantly predicted by vibrato extent but not by vibrato rate (see Supplementary results).

These findings suggest that vibrato extent plays a more prominent role than vibrato rate in expert evaluations of the vibrato quality. The positive association between vibrato extent and perceived vibrato aligns with previous research showing that greater vibrato extent conveys greater emotional expressiveness and vocal maturity (Howes et al., 2004; Prame, 1997). The absence of a vibrato rate effect is likely due to limited variability among singers within the perceptually acceptable range of 5–7 Hz (Järveläinen, 2002).

Because the judges in this study evaluated the entire performance rather than isolated notes, further research is needed to clarify how vibrato rate and vibrato extent influence expert judgments in the context of complete performance.

#### 4.1.2 Resonance and timbre

Previous research (Wapnick and Ekholm, 1997) has found that resonance and timbre are strongly correlated with overall scores. However, in the present study, neither resonance nor timbre significantly predicted the overall scores. One possible explanation is that vibrato parameters, such as rate and extent, may influence resonance and timbre (Manfredi et al., 2015), leading to intertwined evaluations of these vocal attributes (Wooding and Nix, 2016). This overlap may have made vibrato the more dominant factor in the scoring. In Wapnick and Ekholm (1997), the correlation coefficient between vibrato, “color/warm” (a descriptor similar to timbre), and resonance was close to 0.7, and factor analysis grouped these attributes together within the same factor.

Another contributing factor may be the conceptual and perceptual overlap between resonance and timbre. In both vocal pedagogy and auditory-perceptual research, resonance is often considered a subset or acoustic correlate of the broader construct of timbre (Sundberg, 1987). Given this relationship, expert vocal instructors may have found it difficult to consistently differentiate between the two attributes during the evaluation, leading to shared variance and reduced predictive specificity.

In addition, the non-significant effects of resonance and timbre in our study may be partly due to the use of recorded audio rather than live performances. Opera is traditionally performed without microphones, allowing the audience to perceive the singer's natural resonance and timbre as their voice projects throughout the performance space. However, when evaluated through recordings, subtle variations in these qualities may not be fully captured or perceived, because the recording process and playback equipment can alter or mask them (Edwards, 2016; Zoran, 2020). As a result, evaluators may have found it difficult to distinguish between differences in resonance and timbre, leading to a lack of statistical significance in this study. This limitation could be addressed in future research by using high-fidelity, calibrated recording and playback systems designed to preserve the detailed acoustic cues of resonance and timbre.

#### 4.1.3 Diction and intonation

Although diction is often considered crucial in opera performance assessments, it did not strongly influence judges' evaluations in this study. As *Caro mio ben* is commonly taught in Japanese high schools, the participants likely met the minimum standard of Italian pronunciation. This could explain why diction did not significantly affect the overall scores. Furthermore, previous research (Wapnick and Ekholm, 1997) has shown that diction has the lowest correlation with overall scores among various vocal attributes. However, our finding should not imply that diction is unimportant in opera singing. Rather, the non-significant result in the present study likely reflects the limited variability in diction proficiency among participants, who generally demonstrated a uniformly adequate level of pronunciation. This lack of variation may have constrained the model's ability to detect any contribution of diction to the overall evaluation.

Similarly, intonation did not significantly predict the overall scores. Professionally trained singers generally demonstrate a high pitch accuracy and reduced variability in this attribute. Moreover, vibrato, which is frequently employed in opera singing, modulates pitch over extended notes, making precise pitch assessments more challenging (D'Amario et al., 2020). In addition, the limited sample size may have reduced the power of the model to detect statistically significant effects of intonation. With a larger number of participants, subtle pitch deviations may have been more readily captured and reflected in evaluation outcomes.

One possible explanation for the absence of significant effects of diction and intonation is the selection of musical material. *Caro mio ben* was deliberately chosen for its technical simplicity and limited linguistic demands in order to isolate core vocal production skills such as vibrato and resonance. However, this choice may have inadvertently reduced the variability in diction and intonation performance among participants, thereby limiting the statistical power to detect effects related to these attributes.

#### 4.1.4 Expressiveness

Expressiveness did not significantly predict overall scores, possibly because it is a broad and subjective concept and vibrato strongly influences perceived emotional content. Judges may differ in their interpretations of expressiveness, focusing on emotional delivery, phrasing, dynamic shifts, or personal styles. Consequently, these diverse standards could make it more difficult to detect a statistically significant effect once the scores are averaged. Moreover, vibrato is frequently used to convey emotions, including adjustments in rate, extent, duration, and volume (Scherer et al., 2015). Thus, when judges perceive a performance to be highly expressive, they may respond to vibrato, which makes it difficult to isolate expressiveness as a distinct predictor of overall scores.

### 4.2 Effects of acoustic features on overall scores

The linear mixed model regressing the overall scores on acoustic features revealed that a higher SPR was associated with higher overall scores, whereas HNR and LUFS did not show statistically significant effects (Figure 4; Table 6). This finding suggests that singers with a greater difference in power between 2–4 kHz and 0–2 kHz tend to receive higher overall scores.

#### 4.2.1 SPR

SPR emerged as a significant predictor of overall scores, which is consistent with its known role as an indicator of formant structure and vocal projections. Previous studies have suggested that a higher SPR value corresponds to a voice that is perceived as both penetrating and rich in timbre (Watts et al., 2003). In opera, singers must be heard above an orchestra without amplification; therefore, they generally adjust their vocal tract to form singer formants between 2 and 4 kHz to enhance vocal projection (Sundberg, 1987). The higher SPR values associated with such formant tuning suggest that singers with a higher SPR may have achieved better vocal projection, which in turn contributed to their higher overall scores. Moreover, SPR has been shown to correlate with training-related improvements in vocal techniques (Usha et al., 2017), reflecting advanced control of resonance, expiratory pressure, and vocal-fold vibration, which are highly valued in operatic performance.

#### 4.2.2 HNR

HNR is frequently used to evaluate voice quality, clarity, and the ratio of harmonic components to noise (Mouawad et al., 2013). It is also especially helpful in diagnosing voice disorders. However, trained opera singers typically exhibit very little noise in their voices (Ikuma et al., 2022). As a result, the range of HNR values for these singers was relatively small, reducing their usefulness in explaining variations in the overall score. In addition, while HNR captures the degree of “low voice noise,” overall impressions in opera often hinge on factors such as voice resonance, emotional expression, and volume balance. Because HNR primarily measures noise components rather than these expressive elements, it may have had limited impact on overall evaluations. Prior work has also suggested that SPR aligns more closely with subjective evaluations than HNR (Kenny and Mitchell, 2006), further indicating that HNR may play a secondary role in judges' assessments of opera performance.

#### 4.2.3 LUFS

Integrated LUFS is a standardized metric commonly used in audio processing for normalization purposes. It quantifies how loud a signal is on a digital level, averaged over an extended period of time. Previous research has shown that spectral balance and resonance characteristics contribute more to the perceived vocal quality than loudness alone (Collyer et al., 2009). In particular, singer formants, which are concentrated in the 2–4 kHz range, play a critical role in determining how well a voice carries (Sundberg et al., 1993). Emphasizing these frequency components can influence subjective evaluations more strongly than the overall amplitude, which likely explains why the LUFS did not emerge as a significant predictor in the present study.

Increases in vocal intensity are typically accompanied by physiological adjustments (e.g., increased subglottal pressure and changes in vocal tract shaping) that redistribute spectral energy and affect timbre. Therefore, vocal intensity may indirectly influence the perceived vocal quality through these timbral changes. Future research should further explore the relationship between vocal intensity, timbre, and perception of vocal quality.

### 4.3 Insights from the two regression models

This study employed two linear mixed models to predict the overall opera-singing scores. The first model, which focused on the subjective evaluations of vocal attributes, identified vibrato as the most significant predictor (Table 5). The second model, which was based on acoustic characteristics, highlighted SPR as the most significant predictor (Table 6). These findings suggest that both dynamic vocal modulations, represented by vibrato, and spectral balance, represented by SPR, play crucial roles in the evaluation of opera singing.

Vibrato, which is characterized by fluctuations in pitch and amplitude, significantly contributes to a singer's perceived technical sophistication. It enhances the artistic quality of the voice, and listeners often assess a singer's proficiency based on vibrato's rate and extent (Muller et al., 2021). As shown in Figure 2, the top-ranked singer exhibited a stable vibrato (Figure 2A), the middle-ranked singer produced a wider, irregular vibrato (Figure 2B), and the low-ranked singer lacked vibrato entirely (Figure 2C). This is also supported by our analysis based on the acoustic characteristics of vibrato (see Supplementary Figure 1). In opera, well-controlled vibrato frequently enhances emotional depth and dramatic tension, implying that vibrato strongly shapes performance assessment.

Singers with higher SPR values, reflecting an enhanced energy in the 2–4 kHz range, tended to receive higher subjective evaluation scores. The top-ranked singer demonstrated a higher SPR with prominent energy in the 2–4 kHz range (Figure 2A), whereas the lower-ranked singers exhibited a lower SPR (Figure 2C). While this pattern suggests a potential role of SPR in differentiating performance, it should be interpreted with caution, given the limited explanatory power of the acoustic regression model (*R*^2^ = 0.20).

Interestingly, although SPR significantly predicted overall impression scores, judges' explicit ratings of resonance and timbre did not. One possible explanation is that the perceptual qualities of timbre, resonance, and vibrato overlap, which leads to redundancy in the evaluation of these attributes. This interpretation is supported by the VIFs for resonance and timbre (for example VIF = 3.30 for timbre). Such an overlap may have limited the ability of individual perceptual items to emerge as significant predictors, despite their conceptual importance. Alternatively, judges may have been perceptually influenced by spectral energy cues, such as vocal projection or formant clustering, but did not consistently label these qualities as “resonance.”

Taken together, these results suggest that opera-singing evaluations assessed in our dataset of ten singers by four expert listeners depend on both dynamic vocal modulations (such as vibrato) and the spectral structure captured by SPR.

### 4.4 Limitations and future directions

This study has several limitations. The experiment was conducted in a recording environment that did not replicate concert hall acoustics, which may have influenced the assessment of certain vocal qualities, such as resonance and timbre. Future research could explore how different singing environments and acoustic settings affect the evaluations. Moreover, the number of participating singers and judges was limited. While the use of a linear mixed-effects model allowed for valid statistical inferences based on the available data, future studies would benefit from including a larger number of expert judges and singers to improve the generalizability and robustness of the findings. Additionally, the sample was limited to female Japanese singers, which restricts the generalizability of the findings. To enhance the applicability of these results, future studies should include a more diverse participant pool, encompassing singers of various vocal types, male singers, and performers from different cultural backgrounds.

## 5 Conclusion

We found that vibrato had a significant impact on the overall opera performance scores. Moreover, a larger difference between the amplitude peaks in the 0–2 kHz and 2–4 kHz ranges corresponding to a higher SPR was associated with higher scores. These results suggest that vibrato, which reflects dynamic vocal modulation, and SPR, which represents spectral balance, are critical factors for the evaluation of opera singing. The insights from this study can inform vocal training and education by guiding the development of targeted exercises and feedback strategies focused on vibrato and SPR, ultimately fostering more effective improvements in both technical and artistic aspects of singing.

## Data Availability

The raw data supporting the conclusions of this article will be made available by the authors, without undue reservation.
