# Supplementary material for: Perceived vibrato and the singing power ratio explain overall evaluations in opera singing
Source: Front Psychol. 2025 Aug 8;16:1568982. doi: 10.3389/fpsyg.2025.1568982 (PMC12370702; doi:10.3389/fpsyg.2025.1568982)
Supplement: Supplementary file 1 [file Data_Sheet_1.pdf]

frontiers

Supplementary Material

1 Supplementary Method

Vibrato is typically characterized by two acoustic parameters: vibrato rate, which indicates the speed of pitch modulation, and vibrato extent, which reflects the magnitude of pitch deviation from the tonal center (Sundberg, 1995).

Vibrato parameters are not typically derived from entire performances but rather from selected sustained segments. This study also limited its analysis to a specific portion of the piece. Specifically, a B-flat note from the climactic phrase of *Caro mio ben*—the only pitch in the piece sustained for more than one second—was selected. This passage was chosen to provide a consistent and technically meaningful sample of each singer's vibrato performance.

To quantify vibrato characteristics, we calculated vibrato rate and vibrato extent from the fundamental frequency (f_o) contour of the sustained tone using a custom script implemented in MATLAB (R2024a, MathWorks Inc.). This approach, which relies on peak detection in the smoothed f_o contour, follows the method proposed by Howes et al. (2004). The analysis procedure was as follows.

First, the audio signal was read from a monaural .wav file and converted to zero-mean by subtracting its average to remove DC offset. The f_o contour was extracted using the Normalized Cross-correlation Function (NCF) method via the pitch() function in MATLAB, with a window length of 40 ms, an overlap of 30 ms, and a pitch range of 80–1000 Hz. Only valid f_o values (> 0) were retained for further analysis.

The extracted f_o contour was then smoothed using a Savitzky–Golay filter (third-order polynomial, 11-point frame) to reduce microvariations and facilitate peak detection. Local maxima and minima of the smoothed f_o trajectory were detected using the findpeaks() function. Maxima were obtained directly, while minima were extracted by inverting the f_o curve.

Vibrato rate was calculated as the reciprocal of the mean period between successive peaks in the frequency contour:

$$\textit{Vibrato Rate}\ (\text{Hz}) = \frac{1}{\text{mean}(\Delta t_{\text{peaks}})} \qquad \text{(Eq. S1)}$$

where Δt_{peaks} denotes the time intervals (in seconds) between consecutive local maxima in the smoothed frequency trajectory.

Vibrato extent was calculated as the average absolute frequency difference between successive extrema ($\Delta\bar{f}$), then converted to musical cents relative to the estimated mean f_o:

$$\textit{Vibrato extent}\ (\text{cents}) = 1200 \times \log_2((f_o + \Delta\bar{f}) / f_o) \qquad \text{(Eq.S2)}$$

From these sustained-tone segments, we extracted vibrato rate and vibrato extent, and constructed a linear mixed-effects model to predict vibrato evaluation scores, with judge ID included as a random effect to account for inter-rater variability:

$$\textit{Vibrato evaluation score} \sim \textit{Vibrato rate} + \textit{Vibrato extent} + (1 \mid \textit{Judge ID}) \qquad \text{(Eq. S3)}$$

To verify the normality assumption of the residuals, Shapiro–Wilk tests were performed on the model using the shapiro.test() function in R. The result indicated no significant deviation from normality ($W = 0.96$, $p = 0.13$), confirming that the assumption of normality was satisfied.

2 Supplementary Result

Calculated vibrato rate and vibrato extent extracted from each singer's recorded voice are presented in **Supplementary Table 1**. To visually illustrate the differences in vibrato among singers, **Supplementary Figure 1** presents the smoothed f_0 contours of the sustained note for all ten singers, arranged in descending order based on their vibrato evaluation scores.

The results of the linear mixed-effects model (Eq. S3) assessing the influence of vibrato-related acoustic parameters on the subjective evaluation of vibrato are summarized in **Supplementary Table 2**. Among the two predictors, vibrato extent had a significant positive effect on vibrato scores ($\beta = 0.01$, $p = 0.004$). In contrast, vibrato rate did not show a statistically significant effect ($\beta = 0.48$, $p = 0.11$) (**Supplementary Figure 2**). Variance inflation factors (VIFs) for both variables were 1.03.

Both the marginal and conditional R^2 values were 0.22, suggesting that the model's explanatory power stems almost entirely from the fixed effects, with little additional variance accounted for by random effects such as judge ID.

3 Supplementary Figures and Tables

3.1 Supplementary Figures

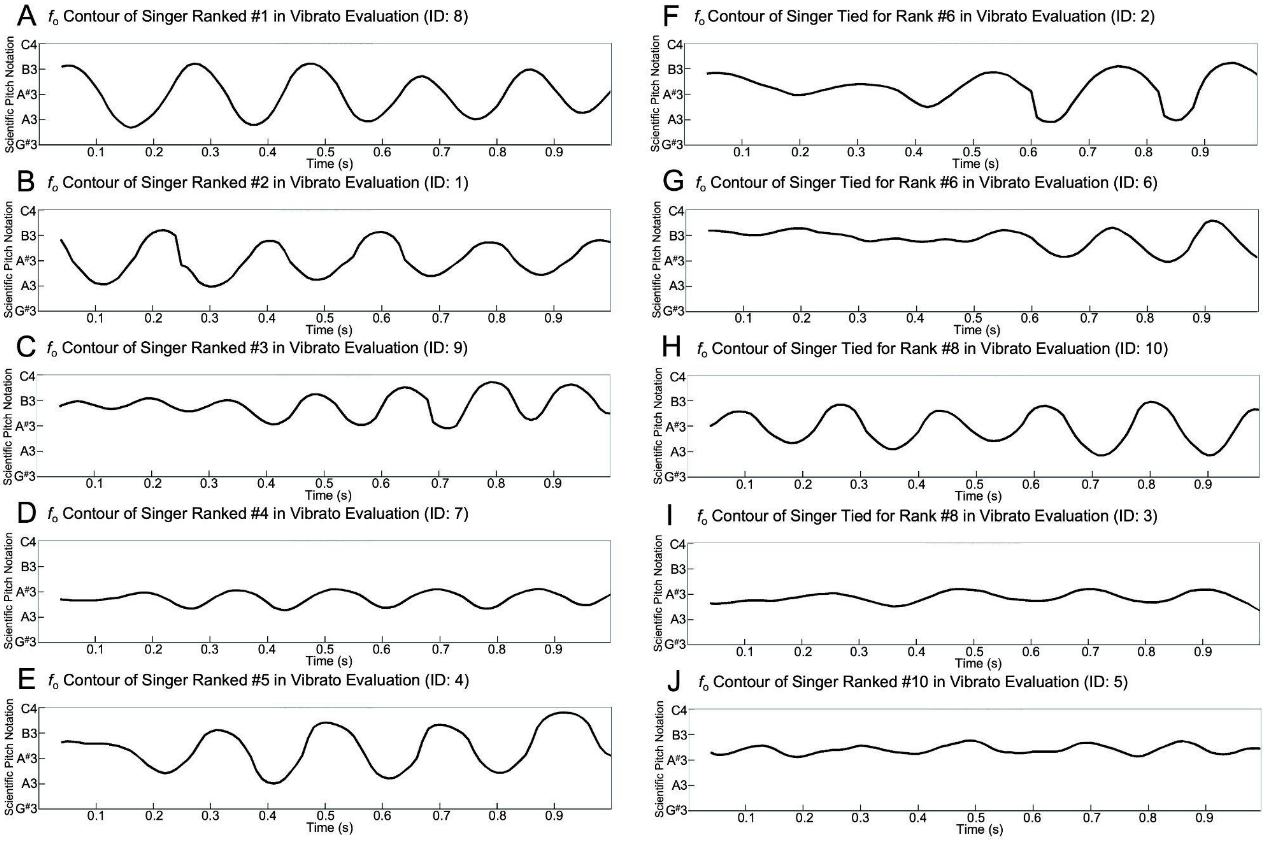

Supplementary Figure 1. f_o contours of ten singers ranked by vibrato evaluation. Each subplot displays the smoothed f_o trajectory of the singer's longest sustained note in *Caro mio ben*. Singers are ordered from highest (**Panel A**) to lowest (**Panel J**) based on their vibrato scores given by expert judges. The y-axis shows scientific pitch notation (G#3 to C4). While higher-ranked singers (Panel **A–C**) exhibit clear, periodic f_o modulations, lower-ranked singers (**Panel H–J**) show reduced or irregular vibrato patterns. **Note**. A# in the figure corresponds to B♭, which is used in the main text to reflect the musical key of the piece.

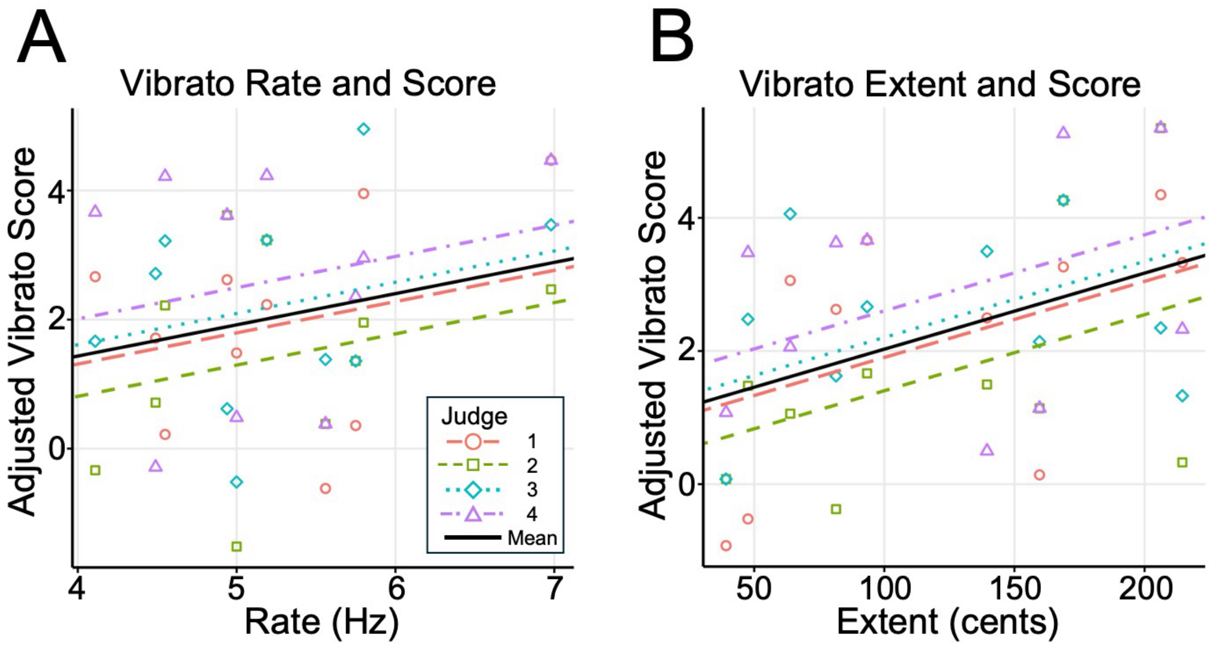

Supplementary Figure 2. Scatter plots showing the relationship between each vibrato parameter and the overall evaluation score, after controlling for the influence of the other parameter. For each panel, a separate linear mixed-effects model was constructed with the target vibrato parameter as a fixed effect and judge ID as a random effect. The adjusted overall score was computed by removing the contribution of the non-target parameter from the original score. **A.** Vibrato rate plotted against the adjusted score. **B.** Vibrato extent against its corresponding adjusted score. Specifically, the adjusted score in Panel A was calculated by subtracting β (vibrato extent) × vibrato extent from the original score, and in Panel B by subtracting β (vibrato rate) × vibrato rate.

3.2 Supplementary Tables

Supplementary Table 1 Calculated Vibrato Parameters from Each Singer's Recorded Voice

ID	Rate (Hz)	Extent (cents)
1	5.19	168.81
2	4.49	139.47
3	4.55	47.60
4	5.00	214.53
5	5.75	39.18
6	4.11	81.45
7	5.80	63.84
8	4.94	206.24
9	6.98	93.38
10	5.56	159.65

Supplementary Table 2 Estimation of Linear Mixed-Effects Models Fitted to Vibrato Score (Fixed Effects: Vibrato Parameters)

Variable	*β*	*SE*	*df*	*t*-value	*p*-value	*VIF*
Rate	0.48	0.29	37.00	1.65	0.11	1.03
Extent	0.01	0.00	37.00	3.05	0.004*	1.03

Note. Asterisk (*) shows statistical significance. Marginal R^2 = 0.22; Conditional R^2 = 0.22.

References

Howes, P., Callaghan, J., Davis, P., Kenny, D., and Thorpe, W. (2004). The relationship between measured vibrato characteristics and perception in Western operatic singing. *J. Voice* 18, 216–230.

Sundberg, J. (1995). Acoustic and psychoacoustic aspects of vocal vibrato. *Vibrato* 35, 35–62.
